# Supplementary material for: Mice with a diverse human T cell receptor repertoire selected on multiple HLA class I molecules
Source: Nat Commun. 2025 Jul 1;16:5432. doi: 10.1038/s41467-025-61306-y (PMC12216589; doi:10.1038/s41467-025-61306-y)
Supplement: Supplementary file 1 — Supplementary Information [file 41467_2025_61306_MOESM1_ESM.pdf]

SUPPLEMENTARY INFORMATION DATA

Mice with a diverse human T cell receptor repertoire selected on multiple HLA class I molecules

Arunraj Dhamodaran[1,2,†], Xiaojing Chen[1], Niklas Fellmer[2], Deepti Agrawal[2], Eric Danner[2], Ralf Kühn[3], Thomas Blankenstein[1*]

Affiliations:

[1]Molecular Immunology and Gene Therapy, Max Delbrück Center for Molecular Medicine in the Helmholtz Association (MDC), Berlin, Germany.

[2]T-knife Therapeutics, Inc., San Francisco, CA.

[3]Genome Engineering and Disease Models, Max Delbrück Center for Molecular Medicine in the Helmholtz Association (MDC), Berlin, Germany.

[†]Present Address: Microbiome and Cancer Division, German Cancer Research Center in the Helmholtz Association (DKFZ), Heidelberg, Germany.

*Corresponding author Email: tblanke@mdc-berlin.de.

CONTENTS

Supplementary Fig. 1. Allele-specific PCR analysis of PiggyBac integrated six-HLA gene cassette in AB*ab*-I mice.

Supplementary Fig. 2. Gating Strategies used for T cell development analysis in AB*ab*-I, AB*ab*-A2, and C57BL6/N mice.

Supplementary Fig. 3. Human HLA class I haplotype expression in murine MCA205 cells.

Supplementary Fig. 4. Gating Strategies used for HLA-I expression analysis, phenotypic characterization of T cells, and functional assessment of $CD8^+$ T cell responses in AB*ab*-I mice.

Supplementary Fig. 5. TLA analysis of PiggyBac integrated six-HLA gene cassette in AB*ab*-I mice.

Supplementary Fig. 6. Absolute T cell counts in F6-F10 generation AB*ab*-I mice versus AB*ab*-A2 mice in comparison to C57BL6/N.

Supplementary Fig. 7. J gene usages in the α and β repertoire of AB*ab*-I and AB*ab*-A2 mice.

Supplementary Fig. 8. V-J pairing frequencies of TCR α and β clonotypes in AB*ab*-I and AB*ab*-A2 mice.

Supplementary Fig. 9. Shared clones among AB*ab*-I and AB*ab*-A2 mice.

Supplementary Fig. 10. TCR recognition of cognate peptide-bound HLAs in AB*ab*-I mice *ex vivo*.

Supplementary Table 1. TCRα and TCRß diversity with in-frame and out-of-frame clonotypes.

Supplementary Table 2. Genotyping PCR primers used for genomic confirmation of all six HLA-I alleles in AB*ab*-I mice.

Supplementary Table 3. Antibodies used for HLA-I expression analysis and T cell characterization assays.

SUPPLEMENTARY FIGURES AND TABLES

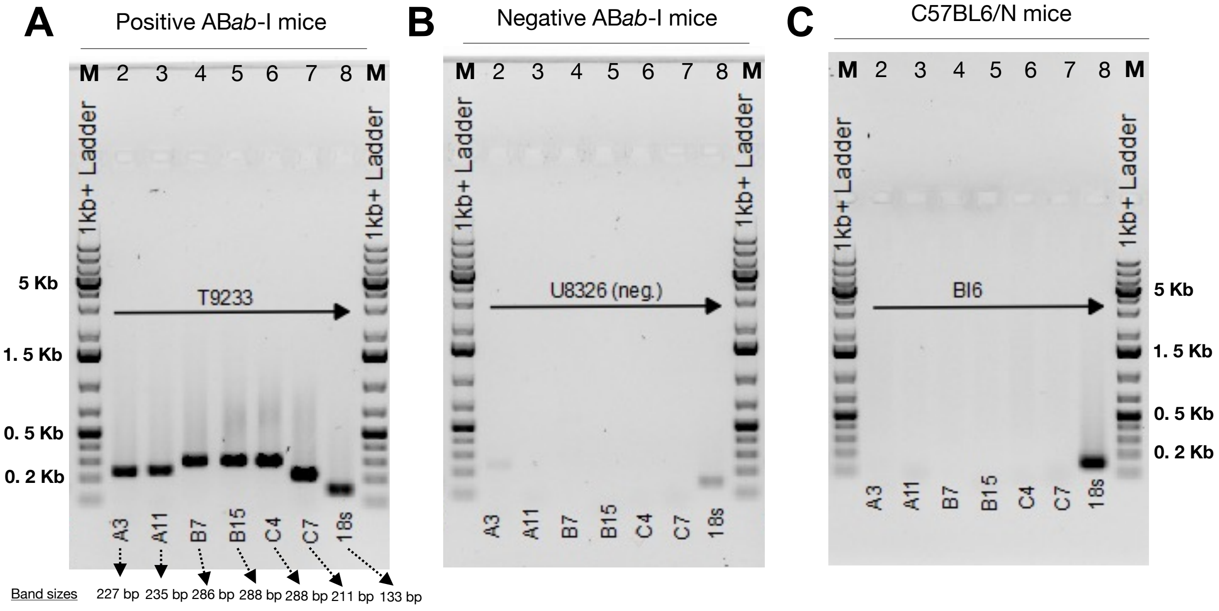

Supplementary Fig. 1. Allele-specific PCR analysis of PiggyBac integrated six-HLA gene cassette in AB*ab*-I mice.

(A) Positive AB*ab*-I mice. PCR-amplified DNA electrophoresis bands show the complete HLA-I haplotype, encompassing six alleles: HLA-*A*03:01*, -*A*11:01*, -*B*07:02*, -*B*15:01*, -*C*04:01*, and -*C*07:02* (Lanes 2-7)**,** with internal control from a stable housekeeping gene, 18S rRNA (Lane 8). (**B)** Negative AB*ab*-I mice and **(C)** C57BL6/N mice. No bands were seen for HLA-I haplotype (Lanes 2-7), but DNA bands were shown for internal control, 18S rRNA reaction (Lane 8). 'M' lanes show O'GeneRuler™ 1 kb plus DNA ladder run. One representative genotyping gel image is shown here from several genotyping assays from over several years of continuous breeding.

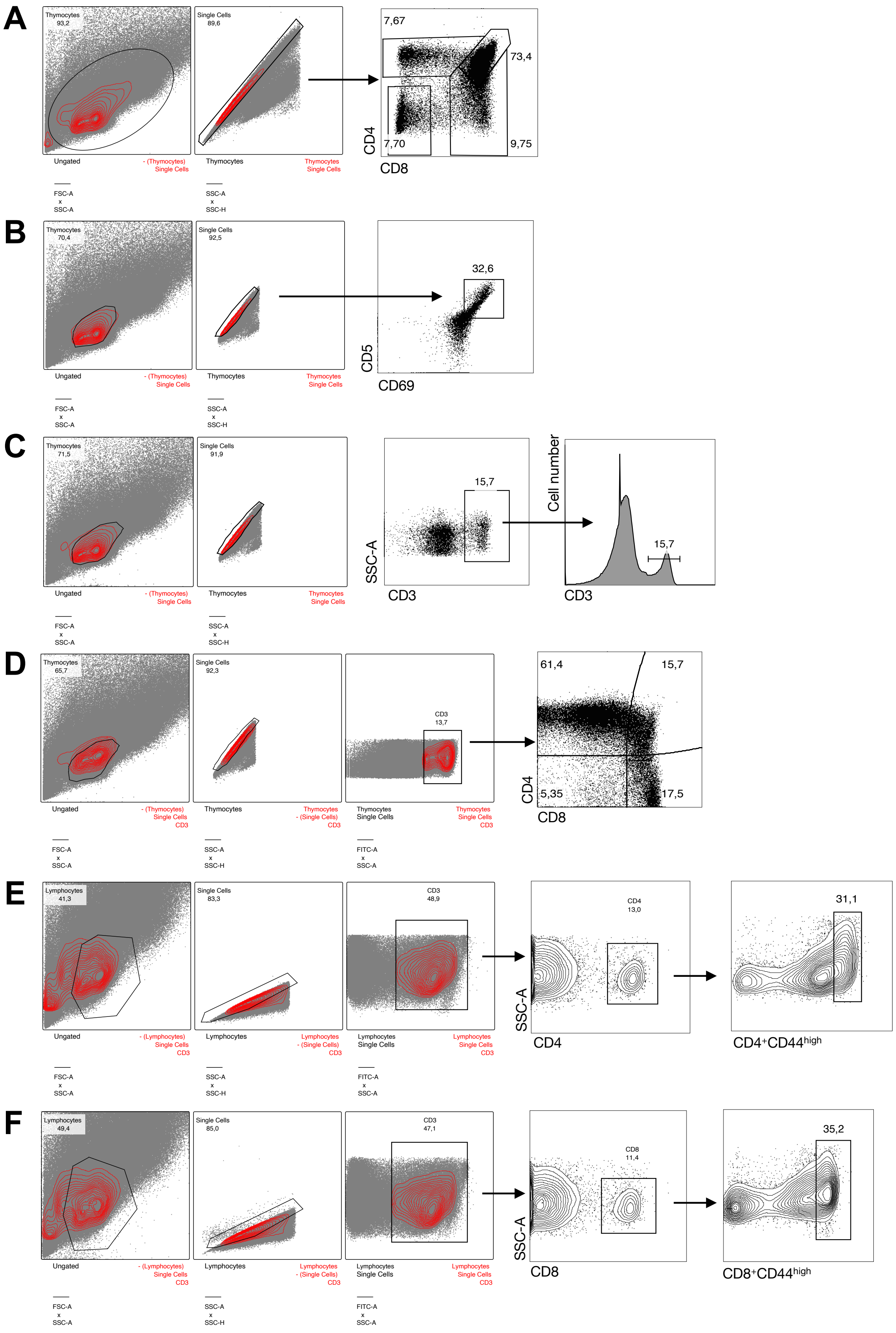

A
Thymocytes
93,2
Single Cells
89,6
7,67
73,4
7,70
9,75
CD4
CD8
Ungated
- (Thymocytes)
Single Cells
Thymocytes
FSC-A
x
SSC-A
SSC-A
x
SSC-H
B
Thymocytes
70,4
Single Cells
92,5
32,6
CD5
CD69
C
Thymocytes
71,5
Single Cells
91,9
15,7
SSC-A
CD3
Cell number
15,7
CD3
D
Thymocytes
65,7
Single Cells
92,3
CD3
13,7
61,4
15,7
5,35
17,5
CD4
CD8
FITC-A
x
SSC-A
E
Lymphocytes
41,3
Single Cells
83,3
CD3
48,9
CD4
13,0
31,1
SSC-A
CD4
CD4+CD44high
F
Lymphocytes
49,4
Single Cells
85,0
CD3
47,1
CD8
11,4
35,2
SSC-A
CD8
CD8+CD44high

Supplementary Fig. 2: Gating Strategies used for T cell development analysis in AB*ab*-I, AB*ab*-A2, and C57BL6/N mice. (A) Flow cytometry gating strategy to delineate $CD4^+$ and $CD8^+$ single- and double-positive thymocyte populations, as presented in the first panel of Fig. 2A. **(B)** Gating strategy to define $CD5^+CD69^+$ thymocyte populations, corresponding to the second panel of Fig. 2A. **(C)** Gating strategy to quantify $CD3^+$ thymocyte populations, shown in the third panel of Fig. 2A. **(D)** Gating strategy to distinguish $CD3^+CD4^+$ and $CD3^+CD8^+$ single- and double-positive thymocytes, as depicted in the fourth panel of Fig. 2A. **(E)** Gating strategy used to quantify $CD4^+CD44^{high}$ splenocytes, corresponding to the second panel of Fig. 2D. **(F)** Gating strategy used to quantify $CD8^+CD44^{high}$ splenocytes, shown in the first panel of Fig. 2D.

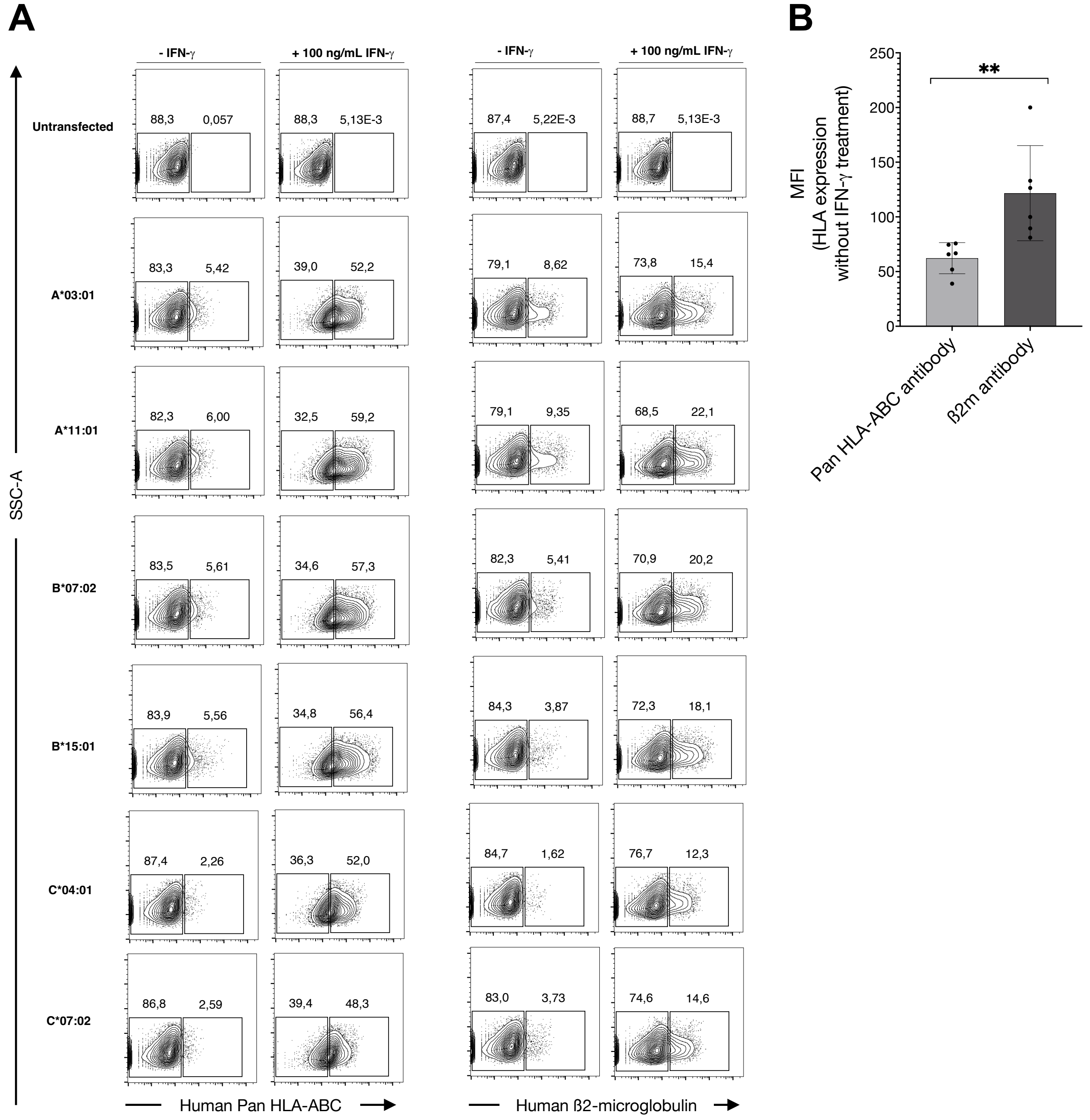

Supplementary Fig. 3. Human HLA class I haplotype expression in murine MCA205 cells. **(A)** Flow cytometry analysis of HLA expression in MCA205 cells transiently transfected with six individual HLA monochains, stained with pan HLA-ABC and ß2m antibodies, with or without 48 hours of IFN-γ treatment. Gated on live cells. One representative plot from multiple independent experiments is shown (n = 3). (**B**) Mean fluorescence intensity (MFI) of HLA expression in transfected MCA205 cells without IFN-γ treatment, stained with pan HLA-ABC or β2m antibodies (n = 6). Each data point represents an independently transfected and analyzed

sample. Statistical comparisons were performed between two groups using a two-tailed unpaired Student's *t*-test. Data in Panel (**B**) are presented as mean ± SD: $**P < 0.01$. Source data are available in the Source Data file.

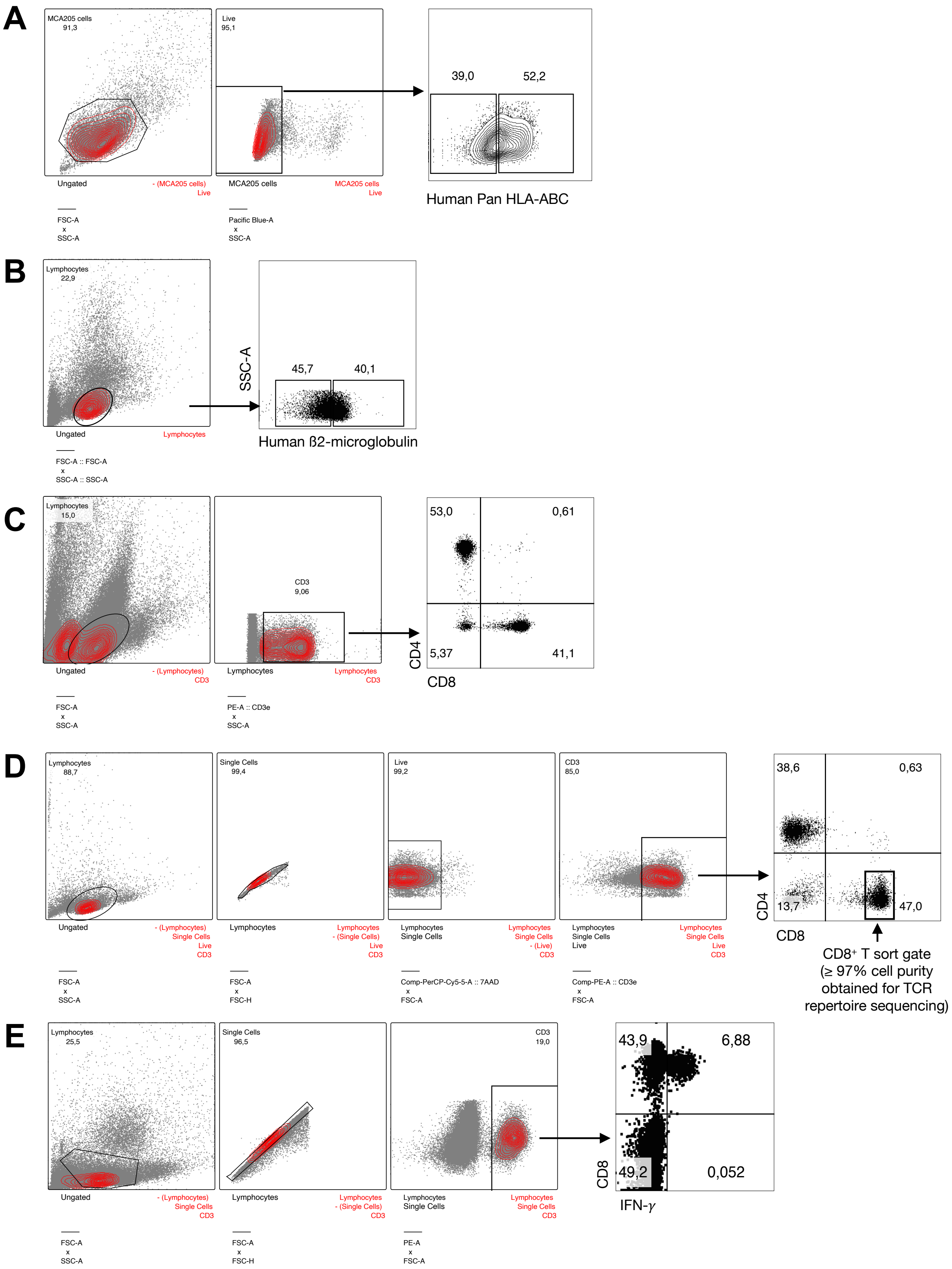

A
MCA205 cells 91,3
Live 95,1
39,0
52,2
Ungated
- (MCA205 cells) Live
MCA205 cells
MCA205 cells Live
FSC-A x SSC-A
Pacific Blue-A x SSC-A
Human Pan HLA-ABC
B
Lymphocytes 22,9
SSC-A
45,7
40,1
Ungated
Lymphocytes
FSC-A :: FSC-A x SSC-A :: SSC-A
Human ß2-microglobulin
C
Lymphocytes 15,0
CD3 9,06
53,0
0,61
5,37
41,1
CD4
CD8
Ungated
- (Lymphocytes) CD3
Lymphocytes
Lymphocytes CD3
FSC-A x SSC-A
PE-A :: CD3e x SSC-A
D
Lymphocytes 88,7
Single Cells 99,4
Live 99,2
CD3 85,0
38,6
0,63
13,7
47,0
CD4
CD8
Ungated
- (Lymphocytes) Single Cells Live CD3
Lymphocytes
Lymphocytes - (Single Cells) Live CD3
Lymphocytes Single Cells
Lymphocytes Single Cells - (Live) CD3
Lymphocytes Single Cells Live
Lymphocytes Single Cells Live CD3
FSC-A x SSC-A
FSC-A x FSC-H
Comp-PerCP-Cy5-5-A :: 7AAD x FSC-A
Comp-PE-A :: CD3e x FSC-A
CD8⁺ T sort gate (≥ 97% cell purity obtained for TCR repertoire sequencing)
E
Lymphocytes 25,5
Single Cells 96,5
CD3 19,0
43,9
6,88
49,2
0,052
CD8
IFN-γ
Ungated
- (Lymphocytes) Single Cells CD3
Lymphocytes
Lymphocytes - (Single Cells) CD3
Lymphocytes Single Cells
Lymphocytes Single Cells CD3
FSC-A x SSC-A
FSC-A x FSC-H
PE-A x FSC-A

Supplementary Fig. 4: Gating Strategies used for HLA-I expression analysis, phenotypic characterization of T cells, and functional assessment of CD8^{+} T cell responses in AB*ab*-I mice. (A) Gating strategy used to quantify individual HLA-I monochains in MCA205 cells stained with pan-HLA-ABC or anti-human ß2m antibodies, as shown in Supplementary Fig. 3A. **(B)** Flow cytometry gating strategy to quantify β2m-associated HLA-I molecules, as shown in the second panel of Fig. 3A. **(C)** Gating strategy to identify CD3^{+}CD8^{+} and CD3^{+}CD4^{+} T cell subsets in peripheral blood, as presented in Fig. 4A and Supplementary Fig. 6A,B. **(D)** Gating strategy to delineate CD3^{+}CD8^{+} and CD3^{+}CD4^{+} T cells in lymphoid organs, as shown in Fig. 4E. The same gating strategy was used to sort CD8^{+} T cells at ≥97% purity for ImmunoSEQ TCR repertoire sequencing, with deep sequencing data presented in Figs. 5–8 and Supplementary Figs. 7–9. **(E)** Gating strategy to assess intracellular IFN-γ expression by CD3^{+}CD8^{+} T cells in peripheral blood of responder mice, as shown in Fig. 10.

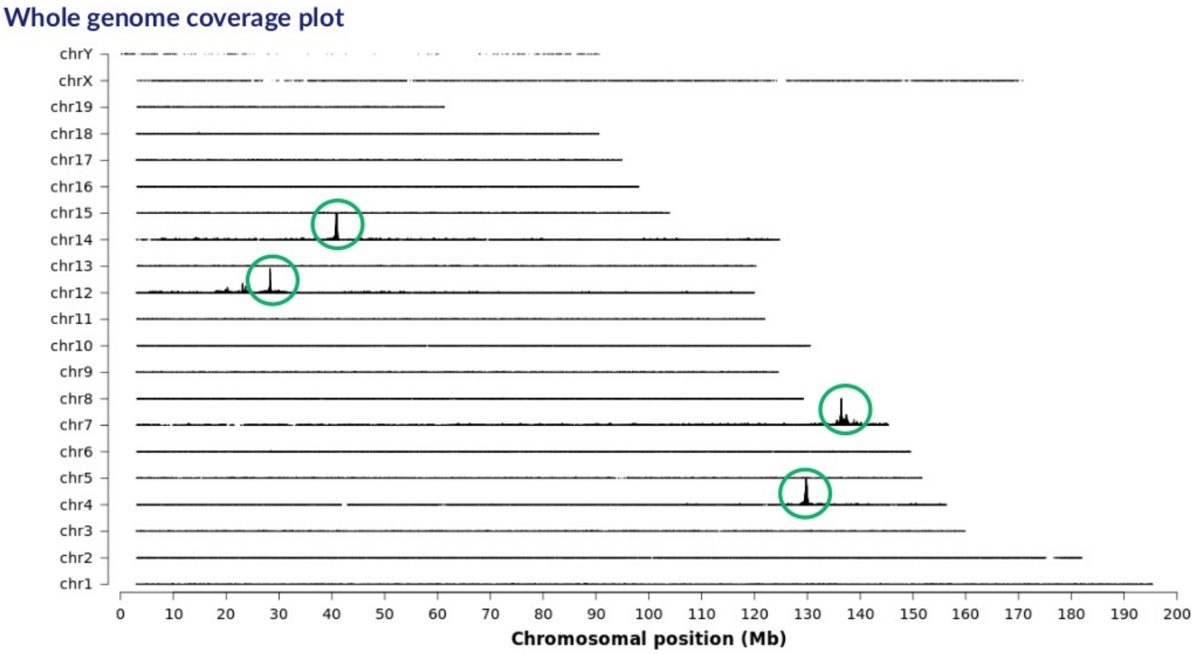

Supplementary Fig. 5. TLA analysis of PiggyBac integrated six-HLA gene cassette in AB*ab*-I mice.

Overview of total coverage of TLA sequencing data showing 4 integration sites of the transgene cassette. Chromosome-specific sequencing performed using genomic DNA from the lymphoid organs of F5 generation AB*ab*-I mice. 4 integration sites include Chr4, Chr7, Chr 12 and Chr 14.

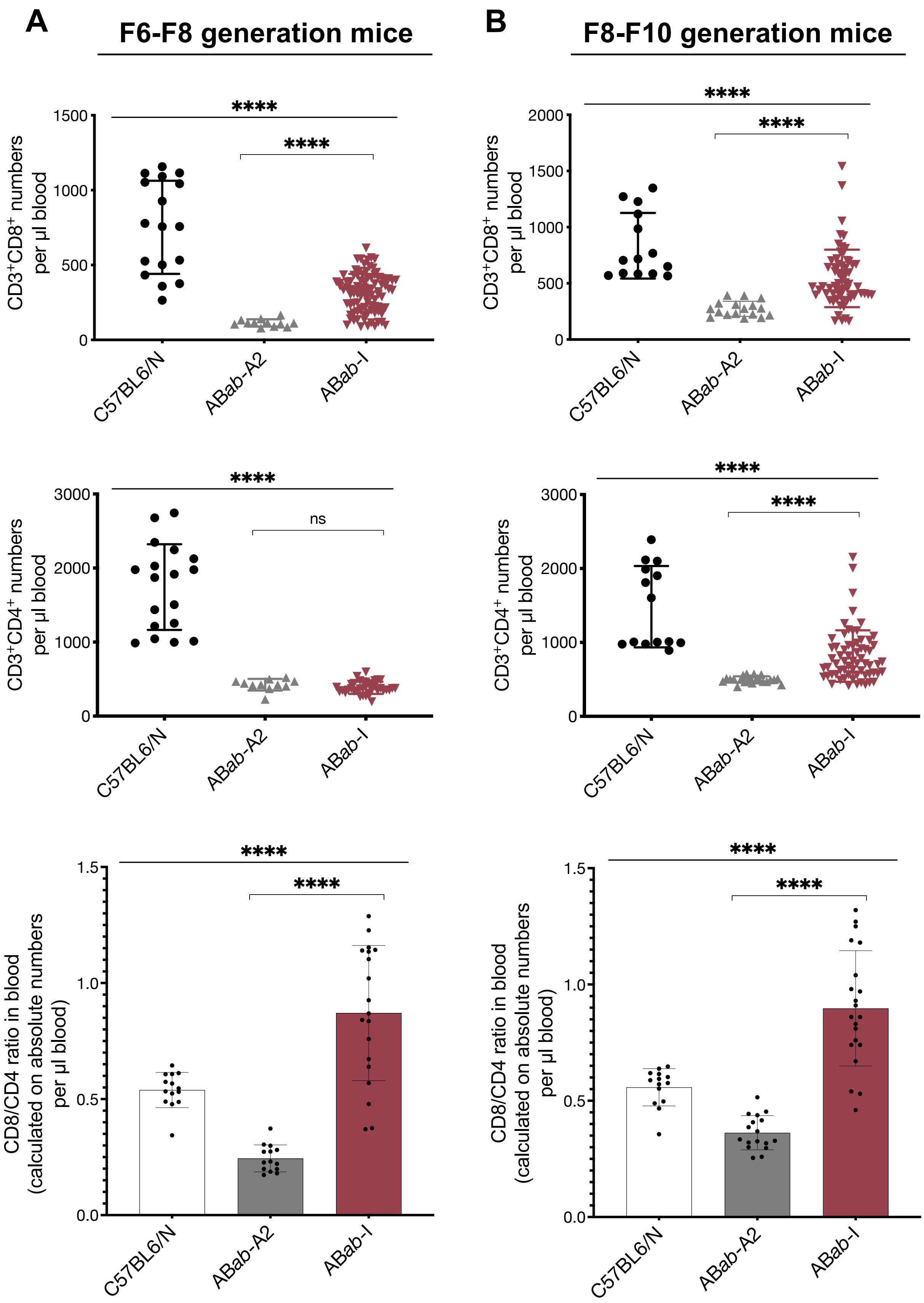
A
F6-F8 generation mice
B
F8-F10 generation mice
CD3+CD8+ numbers per µl blood
CD3+CD4+ numbers per µl blood
CD8/CD4 ratio in blood (calculated on absolute numbers per µl blood)
C57BL6/N
ABab-A2
ABab-I
****
ns

Supplementary Fig. 6. Absolute T cell counts in F6-F10 generation AB*ab*-I mice versus AB*ab*-A2 mice in comparison to C57BL6/N.

(A) AB*ab*-I mice from generations F6 through F8 were monitored for absolute $CD3^+$ $CD8^+$ and $CD4^+$ T cell numbers/µl in peripheral blood with the CD8/CD4 ratio. (**B)** AB*ab*-I mice from generations F8 through F10 were monitored for absolute $CD3^+$ $CD8^+$ and $CD4^+$ T cell numbers/µl in peripheral blood with the CD8/CD4 ratio. Summarized data include C57BL6/N, AB*ab*-A2, and AB*ab*-I mice (n > 10 per group). Each data point represents an individual mouse. Data are presented as mean ± SD. Overall statistical group differences by comparing mutiple groups were assessed by one-way ANOVA, followed by pairwise comparisons using two-tailed unpaired Student's *t*-test. P values indicate: ****P < 0.0001; ns, not significant. Exact P values and source data are available in the Source Data file.

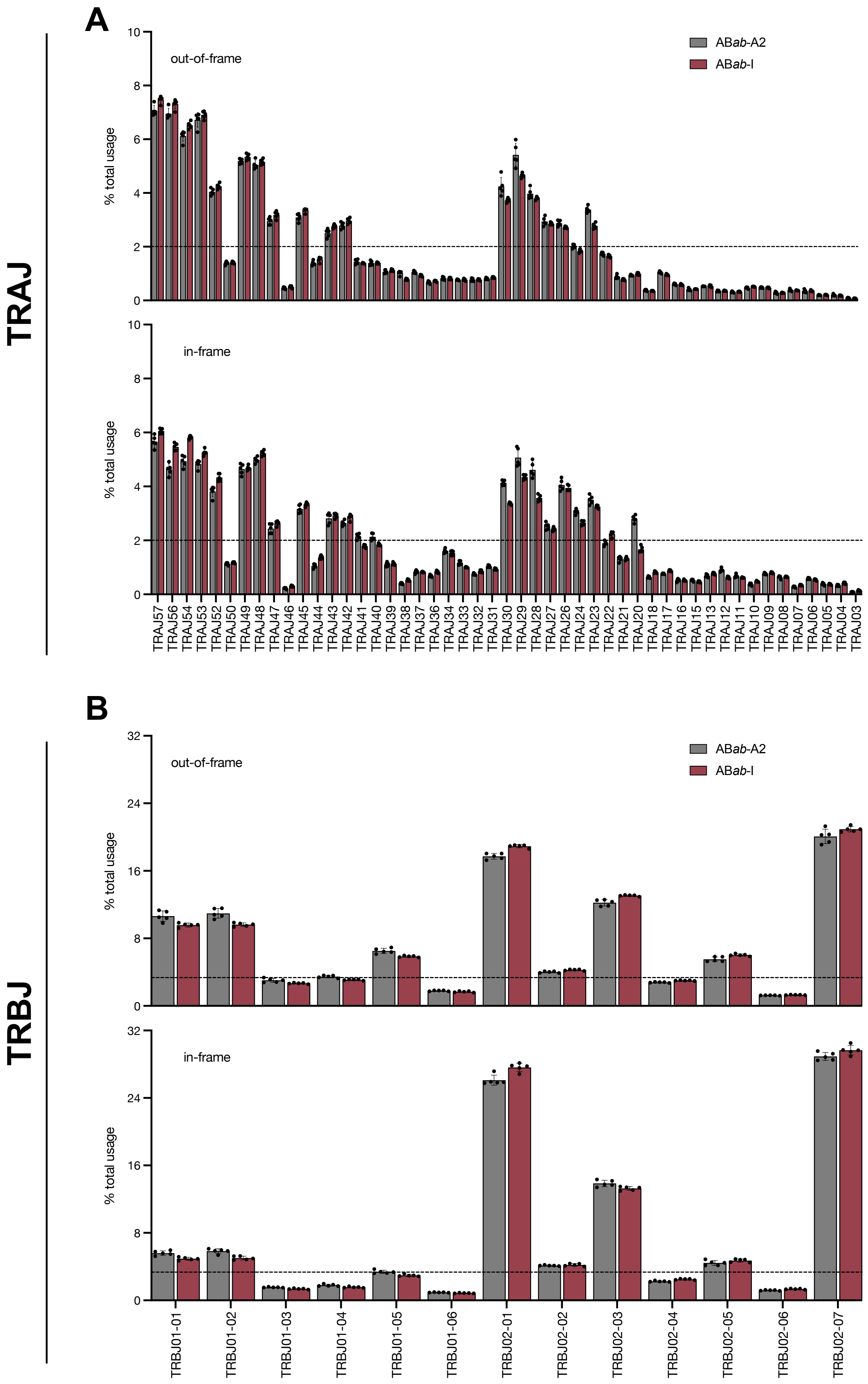
A
TRAJ
out-of-frame
in-frame
ABab-A2
ABab-I
% total usage
0
2
4
6
8
10
TRAJ57
TRAJ56
TRAJ54
TRAJ53
TRAJ52
TRAJ50
TRAJ49
TRAJ48
TRAJ47
TRAJ46
TRAJ45
TRAJ44
TRAJ43
TRAJ42
TRAJ41
TRAJ40
TRAJ39
TRAJ38
TRAJ37
TRAJ36
TRAJ34
TRAJ33
TRAJ32
TRAJ31
TRAJ30
TRAJ29
TRAJ28
TRAJ27
TRAJ26
TRAJ24
TRAJ23
TRAJ22
TRAJ21
TRAJ20
TRAJ18
TRAJ17
TRAJ16
TRAJ15
TRAJ13
TRAJ12
TRAJ11
TRAJ10
TRAJ09
TRAJ08
TRAJ07
TRAJ06
TRAJ05
TRAJ04
TRAJ03
B
TRBJ
out-of-frame
in-frame
ABab-A2
ABab-I
% total usage
0
8
16
24
32
TRBJ01-01
TRBJ01-02
TRBJ01-03
TRBJ01-04
TRBJ01-05
TRBJ01-06
TRBJ02-01
TRBJ02-02
TRBJ02-03
TRBJ02-04
TRBJ02-05
TRBJ02-06
TRBJ02-07

Supplementary Fig. 7. J gene usages in the α and β repertoire of AB*ab*-I and AB*ab*-A2 mice.

Frequencies of J_{α} **(A)** and $J_{ß}$ **(B)** gene usages of unique TCRαß clonotypes in $CD8^{+}$ T cells of AB*ab*-A2 and AB*ab*-I mice. The dotted line represents the frequency of random $J_{\alpha ß}$ gene usage (α, 2.3%; ß, 2.1%). Arrangement of J_{α} and $J_{ß}$ gene segments mentioned on the ***x***-axis according to their position on the human chromosome from 5′ to 3′. 1st top lane: out-of-frame and 2nd bottom lane: in-frame frequencies. Data are from AB*ab*-A2 (n = 5) and AB*ab*-I (n = 5) mice. Data are presented as mean ± SD. Source data are available in the Source Data file.

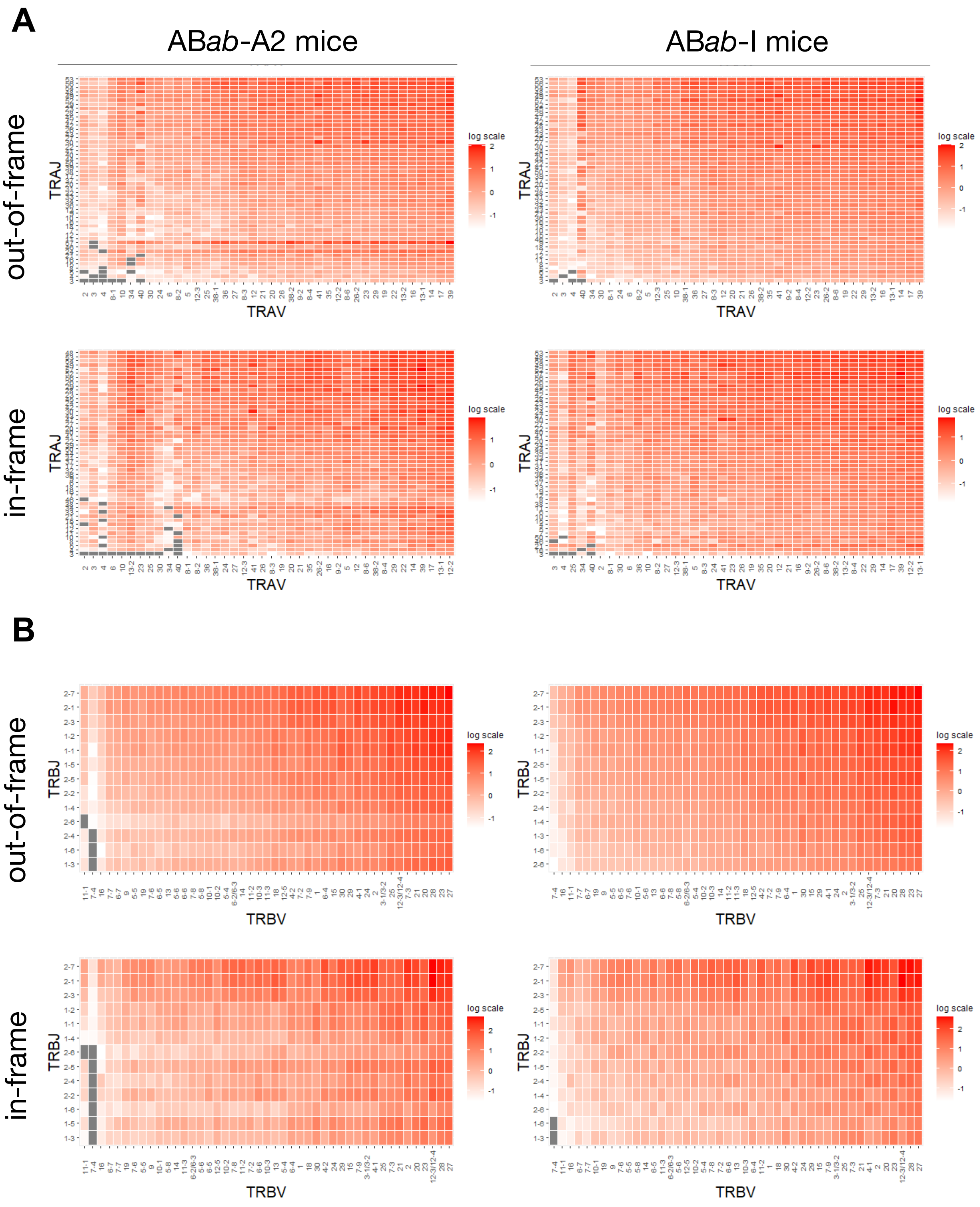
A
ABab-A2 mice
ABab-I mice
out-of-frame
in-frame
TRAJ
TRAV
log scale
B
TRBJ
TRBV

Supplementary Fig. 8. V-J pairing frequencies of TCR α and β clonotypes in AB*ab*-I and AB*ab*-A2 mice.

Mean frequencies of V_{α}-J_{α} **(A)** and V_{β}-J_{β} **(B)** gene pairings of unique TCRαß clonotypes in $CD8^{+}$ T cells of AB*ab*-A2 and AB*ab*-I mice. Arrangement of gene segments (*y*-axis top to bottom, J and *x*-axis left to right, V segments) mentioned are according to their position on the human chromosome from 5′ to 3′. The heat map arrangement represents frequency strength with red to grey (strongest to least). Top lane: in-frame and bottom lane: out-of-frame frequencies. Data are from AB*ab*-A2 (n = 5) and AB*ab*-I (n = 5) mice.

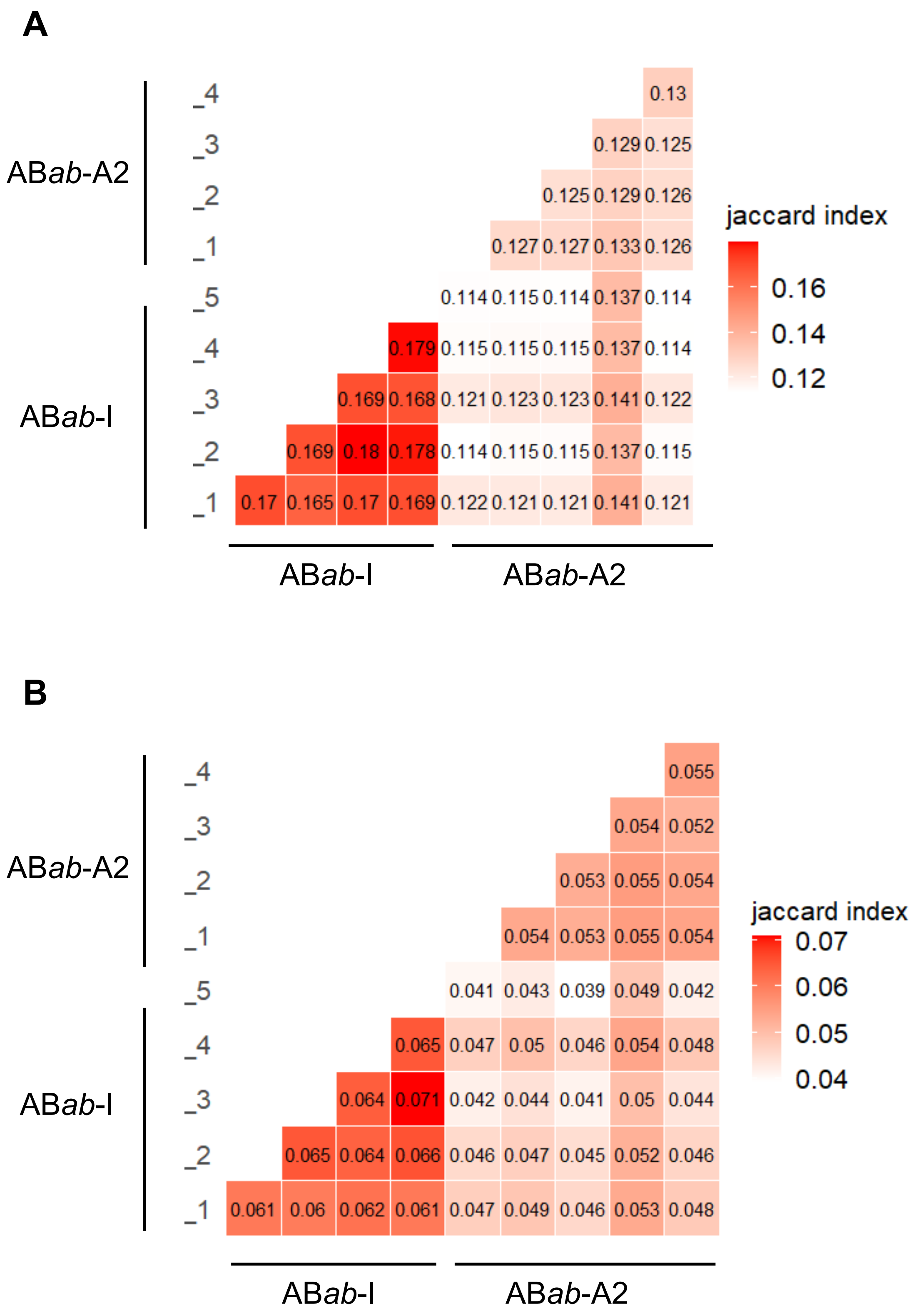
A
ABab-A2
ABab-I
_4
_3
_2
_1
_5
_4
_3
_2
_1
0.13
0.129 0.125
0.125 0.129 0.126
0.127 0.127 0.133 0.126
0.114 0.115 0.114 0.137 0.114
0.179 0.115 0.115 0.115 0.137 0.114
0.169 0.168 0.121 0.123 0.123 0.141 0.122
0.169 0.18 0.178 0.114 0.115 0.115 0.137 0.115
0.17 0.165 0.17 0.169 0.122 0.121 0.121 0.141 0.121
ABab-I
ABab-A2
jaccard index
0.16
0.14
0.12
B
ABab-A2
ABab-I
_4
_3
_2
_1
_5
_4
_3
_2
_1
0.055
0.054 0.052
0.053 0.055 0.054
0.054 0.053 0.055 0.054
0.041 0.043 0.039 0.049 0.042
0.065 0.047 0.05 0.046 0.054 0.048
0.064 0.071 0.042 0.044 0.041 0.05 0.044
0.065 0.064 0.066 0.046 0.047 0.045 0.052 0.046
0.061 0.06 0.062 0.061 0.047 0.049 0.046 0.053 0.048
ABab-I
ABab-A2
jaccard index
0.07
0.06
0.05
0.04

Supplementary Fig. 9. Shared clones among AB*ab*-I and AB*ab*-A2 mice.

Heat map representation of Jaccard index score based on the number of shared TCRα **(A)** and TCRß **(B)** clones within and between groups of AB*ab*-A2 and AB*ab*-I mice represented. The heat map arrangement represents frequency strength with red to grey (strongest to least). Five mice per strain were pooled for analysis.

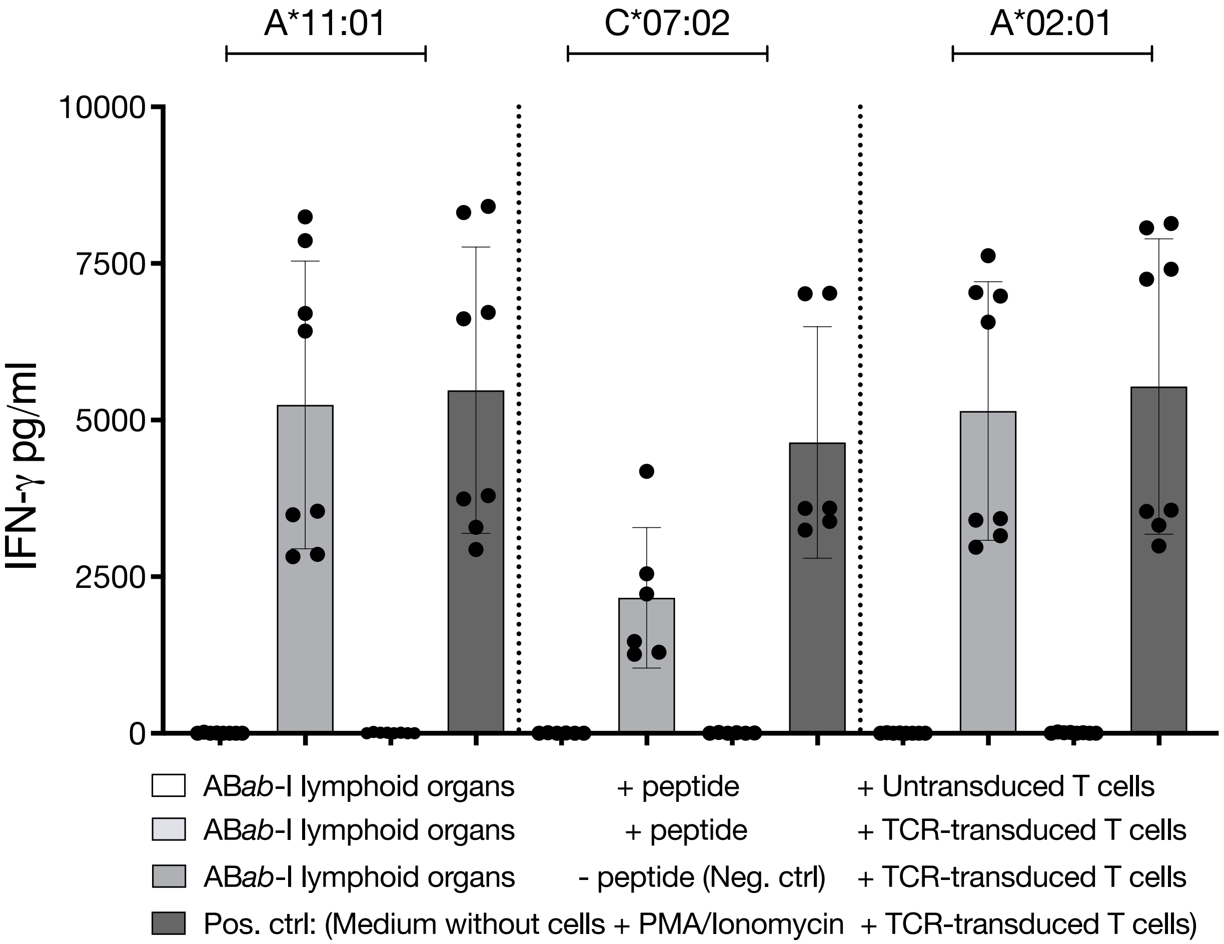

Supplementary Fig. 10. TCR recognition of cognate peptide-bound HLAs in AB*ab*-I mice *ex vivo*.

Ex vivo-isolated lymphoid organs from AB*ab*-I mice were co-cultured with TCR-transduced or untransduced T cells, with or without the addition of cognate peptides. In addition to previously described TCRs, a new TCR specific for the HLA-A2-restricted epitope MAGE-A1$_{278}$ was also tested. Cytokine levels were measured after 16 h of co-culture using a mouse IFN-γ ELISA assay. Consolidated data from multiple TCR co-culture experiments are shown, grouped by HLA restriction as follows: A*11:01, n = 8; C*07:02, n = 6; A*02:01, n = 8. Data are presented as mean ± SD. Source data are available in the Source Data file.

Supplementary Table 1. TCRα and TCRß diversity with in-frame and out-of-frame clonotypes.

The total numbers of in-frame and out-of-frame clonotypes detected in α and ß deep sequencing among AB*ab*-A2 and AB*ab*-I mice with valid total reads. nt, nucleotide, and aa, amino acid clonotypes.

**TCR-α**	**Nr. of inframe seq. (nt)**	**Nr. of inframe seq. (aa)**	**% inframe aa/inframe nt**	**Nr. of inframe (nt) (% of total Nr. (nt))**	**Nr. of out-of-frame (nt)**	**Total reads**
**AB*ab*-A2**	53319	46900	0.879611	0.38446	196246	362147
	56107	49367	0.879872	0.392271	240095	389542
	54829	48554	0.885553	0.37694	201305	339300
	77765	67065	0.862406	0.382807	253578	433140
	54534	48275	0.885228	0.382079	255208	366840
**AB*ab*-I**	122573	102394	0.835372	0.398108	80612	822804
	154974	126692	0.817505	0.389378	87987	821040
	121786	101915	0.836837	0.383817	88023	674599
	157279	128529	0.817204	0.381586	126029	913362
	157803	128726	0.815739	0.38589	86786	903633

**TCR-ß**	**Nr. of inframe seq. (nt)**	**Nr. of inframe seq. (aa)**	**% inframe aa/inframe nt**	**Nr. of inframe (nt) (% of total Nr. (nt))**	**Nr. of out-of-frame (nt)**	**Total reads**
**AB*ab*-A2**	46197	42897	0.92856679	0.477582161	50534	207469
	52844	49075	0.92867686	0.450207451	64533	270263
	42987	40072	0.932188801	0.466610946	49139	201766
	67441	61911	0.918002402	0.460162801	79118	286749
	49214	45742	0.929450969	0.472094853	55032	253233

TCR-ß	Nr. of inframe seq. (nt)	Nr. of inframe seq. (aa)	% inframe aa/inframe nt	Nr. of inframe (nt) (% of total Nr. (nt))	Nr. of out-of-frame (nt)	Total reads
**AB*ab*-I**	90804	83512	0.919695168	0.463775518	104989	386697
	112554	102863	0.913899106	0.465901988	129029	420213
	139591	126336	0.905044021	0.475235761	154139	400974
	105319	96018	0.91168735	0.471766318	117925	369917
	170114	152425	0.896016789	0.481303969	183330	446095

Supplementary Table 2. Genotyping PCR primers used for genomic confirmation of all six HLA-I alleles in AB*ab*-I mice.

Primers	Sequence
**Primers used for HLA-*A*03:01* PCR reaction (product length - 227 bp)**	
GP-A3-1	ACCGACAGAGTGGACCTGG
GP-A3-2	TCCCACTTTCTCTTGGTGATCTGG
**Primers used for HLA-*A*11:01* PCR reaction (product length - 235 bp)**	
GP-A11-3	CAAAGCTCAGTCCCAGACAGAC
GP-A11-4	CCGCTTTGTAATCTGAGCAGC
**Primers used for HLA-*B*07:02* PCR reaction (product length - 286 bp)**	
GP-B7-5	CCCAGATCTACAAGGCTCAGGCC
GP-B7-6	TAGGCCCTTCTCTGCTCAGC
**Primers used for HLA-*B*15:01* PCR reaction (product length - 288 bp)**	
GP-B15-7	CAACACCCAGACATACAGAGAGTCC
GP-B15-8	ACGCACAGTCCTTCCAGATAGG
**Primers used for HLA-*C*04:01* PCR reaction (product length - 288 bp)**	
GP-C4-9	CCCAGAAGTACAAGAGACAGGCC
GP-C4-10	GATAGGCTCTGCGCTGTTCG
**Primers used for HLA-*C*07:02* PCR reaction (product length - 211 bp)**	
GP-C7-11	GAACCTAGATTCATCAGCGTCGG
GP-C7-12	CGGACTGGTTATAGTAGCCTCTGAG
**Primers used for *18s rRNA* - control PCR reaction (product length - 133 bp)**	
18s rRNA For	GGCCGTTCTTAGTTGGTGGAGCG
18s rRNA Rev	CTGAACGCCACTTGTCCCTC

Supplementary Table 3. Antibodies used for HLA-I expression analysis and T cell characterization assays.

Antibody	Catalog Number	Clone	Dilution	Application in this study	Validation in Peer-Reviewed Articles
Anti-mouse CD16/32 Fc receptor block	BioLegend, 101320	93	1:100	Fc receptor block for T cell staining	Poncette et al., 2019, J Clin Invest.
Anti-mouse CD28 (unconjugated for cell culture)	BioLegend, 102116	37.51	0.1 µg/ml	Used in T cell activation assays	Leisegang et al., 2016, Clin Cancer Res.; Engels et al., 2012, Mol Ther
Anti-mouse CD3 (unconjugated for cell culture)	BioLegend, 100340	OKT-3	1 µg/ml	T cell activation for transduction	Leisegang et al., 2016, Clin Cancer Res.; Engels et al., 2012, Mol Ther
Anti-mouse CD3ε (PE)	BioLegend, 100308	145-2C11	1:200	For intracellular staining of T cells	Obenaus et al., 2015, Nat Biotechnol.
Anti-mouse CD8α (APC)	BioLegend, 100712	53-6.7	1:200	For flow cytometry of CD8+ T cells	Obenaus et al., 2015, Nat Biotechnol.
Anti-mouse IFN-γ (BV421)	BioLegend, 505830	XMG1.2	1:200	For IFN-γ cytokine detection in T cells	Obenaus et al., 2015, Nat Biotechnol.
Anti-mouse CD4 (BV421)	BioLegend, 100438	GK1.5	1:200	For T cell subset characterization	Chen et al., 2017, J Exp Med.

**Antibody**	**Catalog Number**	**Clone**	**Dilution**	**Application in this study**	**Validation in Peer-Reviewed Articles**
Anti-mouse CD44 (FITC)	BioLegend, 156007	NIMR8	1:200	For T cell activation and memory markers	Chen et al., 2017, J Exp Med.
Anti-mouse CD5 (PE/Cy7)	BioLegend, 100621	53-7.3	1:200	For T cell subset analysis	Chen et al., 2017, J Exp Med.
Anti-mouse CD69 (PE)	BioLegend, 104507	H1.2F3	1:200	For early T cell activation marker	Chen et al., 2017, J Exp Med.
Anti-human ß2 microglobulin (PE)	BD Pharmingen, 551337	TÜ99	1:200	For human β2m detection in flow cytometry	Boucherma et al., 2014, J Immunol.
Anti-human Pan HLA-ABC (PE)	BioLegend, 311406	W6/32	1:100	For human HLA-I expression analysis	Gavvovidis et al., 2018, Clin Cancer Res.; Shields and Ribaudo, 1998, Tissue Antigens)
