# Supplementary material for: Mice with a diverse human T cell receptor repertoire selected on multiple HLA class I molecules
Source: Nat Commun. 2025 Jul 1;16:5432. doi: 10.1038/s41467-025-61306-y (PMC12216589; doi:10.1038/s41467-025-61306-y)
Supplement: Supplementary file 2 — Reporting Summary [file 41467_2025_61306_MOESM2_ESM.pdf]

nature portfolio

Corresponding author(s): Prof. Dr. Thomas Blankenstein

Last updated by author(s): May 31, 2025

# Reporting Summary

Nature Portfolio wishes to improve the reproducibility of the work that we publish. This form provides structure for consistency and transparency in reporting. For further information on Nature Portfolio policies, see our Editorial Policies and the Editorial Policy Checklist.

## Statistics

For all statistical analyses, confirm that the following items are present in the figure legend, table legend, main text, or Methods section.

| n/a | Confirmed | |
|---|---|---|
| ☐ | ☒ | The exact sample size (*n*) for each experimental group/condition, given as a discrete number and unit of measurement |
| ☐ | ☒ | A statement on whether measurements were taken from distinct samples or whether the same sample was measured repeatedly |
| ☐ | ☒ | The statistical test(s) used AND whether they are one- or two-sided<br>*Only common tests should be described solely by name; describe more complex techniques in the Methods section.* |
| ☒ | ☐ | A description of all covariates tested |
| ☐ | ☒ | A description of any assumptions or corrections, such as tests of normality and adjustment for multiple comparisons |
| ☐ | ☒ | A full description of the statistical parameters including central tendency (e.g. means) or other basic estimates (e.g. regression coefficient) AND variation (e.g. standard deviation) or associated estimates of uncertainty (e.g. confidence intervals) |
| ☐ | ☒ | For null hypothesis testing, the test statistic (e.g. *F*, *t*, *r*) with confidence intervals, effect sizes, degrees of freedom and *P* value noted<br>*Give P values as exact values whenever suitable.* |
| ☒ | ☐ | For Bayesian analysis, information on the choice of priors and Markov chain Monte Carlo settings |
| ☒ | ☐ | For hierarchical and complex designs, identification of the appropriate level for tests and full reporting of outcomes |
| ☒ | ☐ | Estimates of effect sizes (e.g. Cohen's *d*, Pearson's *r*), indicating how they were calculated |

*Our web collection on statistics for biologists contains articles on many of the points above.*

## Software and code

Policy information about availability of computer code

| | |
|---|---|
| Data collection | The original human HLA sequences used in this study for the chimeric ABab-I transgene construction were retrieved from the Immunogenetics (IMGT)/HLA sequence database (https://www.ebi.ac.uk/ipd/imgt/hla/allele.html).<br><br>HLA allele frequencies and ranking information for the top six most frequent HLA I alleles were collected from open-source repositories:<br>1. Allele Frequency Net Database (http://allelefrequencies.net)<br>2. National Marrow Donor Program (NMDP) Bioinformatics (https://bioinformatics.bethematchclinical.org/hla-resources/haplotype-frequencies/high-resolution-hla-alleles-and-haplotypes-in-the-us-population/)<br><br>Global cancer incidence data were obtained from the Global Cancer Observatory (GLOBOCAN) dataset (http://globocan.iarc.fr) |

| | |
|---|---|
| Data analysis | Prediction of peptide-MHC class I binding was performed using artificial neural networks (ANNs) and stabilized matrix methods (SMMs) from the following web-based open-source algorithms:<br>1. NetMHC: https://services.healthtech.dtu.dk/services/NetMHC-4.0/<br>2. IEDB: https://www.iedb.org/home_v3.php<br><br>Deep-sequenced TCR repertoire sequences are stored in the ImmuneSEQ Analyzer database of Adaptive Biotechnologies Inc., Seattle (USA). The TCRαß repertoire deep sequencing data have been deposited in the ImmuneAccess database by Adaptive Biotechnologies and can be accessed under the following DOI links: [TCRα: https://doi.org/10.21417/AD2025NC1; TCRß: https://doi.org/10.21417/AD2025NC2].<br>Statistical analysis was performed using the R program, as described by Chen et al. (2017). |

For manuscripts utilizing custom algorithms or software that are central to the research but not yet described in published literature, software must be made available to editors and reviewers. We strongly encourage code deposition in a community repository (e.g. GitHub). See the Nature Portfolio guidelines for submitting code & software for further information.

# Data

Policy information about availability of data

All manuscripts must include a data availability statement. This statement should provide the following information, where applicable:

- Accession codes, unique identifiers, or web links for publicly available datasets
- A description of any restrictions on data availability
- For clinical datasets or third party data, please ensure that the statement adheres to our policy

Model TCRs restricted to known epitopes presented by HLA alleles in ABab-I mice were obtained from the original publication accessions cited in this study for in vivo immunogenicity validation.

The TCRαß repertoire deep sequencing data have been deposited in the ImmuneAccess database by Adaptive Biotechnologies and can be accessed under the following DOI links: [TCRα: https://doi.org/10.21417/AD2025NC1; TCRß: https://doi.org/10.21417/AD2025NC2].

# Research involving human participants, their data, or biological material

Policy information about studies with human participants or human data. See also policy information about sex, gender (identity/presentation), and sexual orientation and race, ethnicity and racism.

| | |
|---|---|
| Reporting on sex and gender | n/a |
| Reporting on race, ethnicity, or other socially relevant groupings | n/a |
| Population characteristics | n/a |
| Recruitment | n/a |
| Ethics oversight | n/a |

Note that full information on the approval of the study protocol must also be provided in the manuscript.

# Field-specific reporting

Please select the one below that is the best fit for your research. If you are not sure, read the appropriate sections before making your selection.

- [x] Life sciences
- [ ] Behavioural & social sciences
- [ ] Ecological, evolutionary & environmental sciences

For a reference copy of the document with all sections, see nature.com/documents/nr-reporting-summary-flat.pdf

# Life sciences study design

All studies must disclose on these points even when the disclosure is negative.

| | |
|---|---|
| Sample size | The sample size for this study was based on established practices in immunology for TCR repertoire analysis. We used 6-7 mice per group for immunizations, consistent with studies by Li et al. (2010) and Chen et al. (2017), which showed this range is adequate for achieving statistically significant results and robust comparisons. Preliminary experiments indicated that this sample size adequately represents biological variability and allows for meaningful detection of differences in TCR diversity and immune response against tumor antigens. No formal sample size calculation was performed, as the design was driven by the study's objectives and supported by prior work. |
| Data exclusions | We included all reads with V genes identified as TRBV20, unlike our previous work by Chen et al. (2017), which counted only TRBV20-01 chains. The frequency of random Vαß gene usage was set with cut-offs of 2.3% for TRAV and 2.1% for TRBV. The expression of Vαß genes TRAV01-01, TRBV05-01, and TRBV06-01 was absent in both ABab-A2 and ABab-I mice.<br><br>TRAV18 usage was not detected, likely due to PCR bias from the ImmunoSEQ platform, and was excluded from Vαß frequency usage graphs. |

| | |
|---|---|
| Replication | All experiments were successfully reproduced from in vitro validation through to mouse generation, with one representative example shown for each independently reproduced experiment, indicated by 'n' in the figure legends. Different founders of the ABab-I mice were thoroughly analyzed for reproducible results related to robust immune responses. Throughout the study, we used 8- to 20-week-old mice for characterization experiments (at least 5 mice/group, 6-7 mice/group for immunizations). Five mice per group per strain were sent for TCR repertoire deep sequencing; no independent repetition was necessary for repertoire diversity assessment. Reproducibility is further supported by data that aligns with previous work by Chen et al. (2017) |
| Randomization | Samples were allocated into experimental groups randomly to minimize bias in the analysis. For mouse experiments, both genders were included in equal numbers across groups to ensure a balanced representation and enhance the reliability of the results. |
| Blinding | Blinding was not necessary for this study, as the objective nature of the immune response measurements and TCR analysis minimizes bias. The results were reproducible and aligned with previous findings by Li et al. (2010), Poncette et al. (2019), and Chen et al. (2017), further supporting the reliability of the data. |

# Reporting for specific materials, systems and methods

We require information from authors about some types of materials, experimental systems and methods used in many studies. Here, indicate whether each material, system or method listed is relevant to your study. If you are not sure if a list item applies to your research, read the appropriate section before selecting a response.

## Materials & experimental systems

| n/a | Involved in the study |
|---|---|
| ☐ | ☒ Antibodies |
| ☐ | ☒ Eukaryotic cell lines |
| ☒ | ☐ Palaeontology and archaeology |
| ☐ | ☒ Animals and other organisms |
| ☒ | ☐ Clinical data |
| ☒ | ☐ Dual use research of concern |
| ☒ | ☐ Plants |

## Methods

| n/a | Involved in the study |
|---|---|
| ☒ | ☐ ChIP-seq |
| ☐ | ☒ Flow cytometry |
| ☒ | ☐ MRI-based neuroimaging |

## Antibodies

Antibodies used

Antibodies utilized in this study include:

Anti-mouse CD16/32 Fc receptor block (BioLegend, Catalog Number: 101320, Clone: 93).
Anti-mouse CD28 (unconjugated for cell culture) (BioLegend, Catalog Number: 102116, Clone: 37.51).
Anti-mouse CD3 (unconjugated for cell culture) (BioLegend, Catalog Number: 100340, Clone: OKT-3).
Anti-mouse CD3ε (PE) (BioLegend, Catalog Number: 100308, Clone: 145-2C11).
Anti-mouse CD8α (APC) (BioLegend, Catalog Number: 100712, Clone: 53-6.7).
Anti-mouse IFN-γ (BV421) (BioLegend, Catalog Number: 505830, Clone: XMG1.2).
Anti-mouse CD4 (BV421) (BioLegend, Catalog Number: 100438, Clone: GK1.5).
Anti-mouse CD44 (FITC) (BioLegend, Catalog Number: 156007, Clone: NIMR8).
Anti-mouse CD5 (PE/Cy7) (BioLegend, Catalog Number: 100621, Clone: 53-7.3).
Anti-mouse CD69 (PE) (BioLegend, Catalog Number: 104507, Clone: H1.2F3).
Anti-human ß2 microglobulin (PE) (BD Pharmingen, Catalog Number: 551337, Clone: TÜ99).
Anti-human Pan HLA-ABC antibody (PE) (BioLegend, Catalog Number: 311406, Clone: W6/32).

Validation

All antibodies were validated and QC tested for either flow cytometry or murine T cell transduction applications. The antibodies were sourced from previously published peer-reviewed articles:

The anti-mouse CD16/32 antibody was validated for reactivity against mouse Fc receptors, as described in the intracellular T cell staining protocol by Poncette et al. (2015, J Clin Invest.) Dilution: 1:100.

The anti-mouse CD28 and anti-mouse CD3 antibodies were adopted from murine T cell transduction protocols described by Leisegang et al. (2016, Clin Cancer Res), and Engels et al. (2012, Mol Ther), and were validated for reactivity against mouse CD28 and CD3 chains. Concentration: 0.1 µg/ml (CD28), 1 µg/ml (CD3).

The anti-mouse CD3ε, anti-mouse CD8α , and anti-mouse IFN-γ antibodies were adopted from the intracellular T cell response assessment protocol by Obenaus et al. (2015, Nat Biotechnol). These antibodies were titrated and validated for reactivity against CD3ε and CD8α chains, and dimerized IFN-γ receptors. Dilution: 1:200.

The anti-mouse CD4, CD44, CD5, and CD69 antibodies were adopted from the T cell development staining protocol described by Chen et al. (2017, J Exp Med). These antibodies were validated to bind mouse CD4, CD44, CD5, and CD69 chains. Dilution: 1:200.

The anti-human ß2 microglobulin antibody was adopted from the murine splenocyte staining protocol described by Boucherma et al. (2014, J Immunol). This antibody was validated for reactivity against human complexed or free ß2-microglobulin, and clone TÜ99

does not react with ß2-microglobulin from other species. Dilution: 1:200.

The anti-human Pan HLA-ABC antibody was validated for reactivity against HLA class I molecules in human cell lines as described in the study by Gavvovidis et al. (2018, Clin Cancer Res.). This antibody has been extensively used in flow cytometry to assess HLA-I expression on various cell types. Additionally, the reactivity of the W6/32 antibody was demonstrated in the study by Shields and Ribaudo (1998, Tissue Antigens), where it was used for staining acid-stripped cells expressing recombinant or native human ß2m. Dilution used in this study: 1:100.

## Eukaryotic cell lines

Policy information about cell lines and Sex and Gender in Research

| | |
|---|---|
| Cell line source(s) | To verify surface expression of individual HLA-I monochains, we used MCA205 murine fibrosarcoma cell line.<br>All other cells used in this study were primary materials from transgenic mice:<br>ABab-A2/ABabDII mice, as described by Li et al. (2010, Nat Medicine);<br>C57BL6/N mice, bred in-house at the MDC core facility in Berlin, Germany;<br>ABab-I mice, as described in this study. |
| Authentication | All cells used in this study were primary materials derived from transgenic mice, except for the MCA205 murine cell line used in transfection experiments shown in Supplementary Fig. 3A. MCA205 cells were authenticated by flow cytometry to confirm the absence of endogenous human HLA expression—unstained controls showed no signal—ensuring they were suitable hosts for the surface expression of our individual human HLA-I monochain constructs. This allowed us to specifically assess the expression of introduced HLA alleles. No additional authentication methods were performed.<br>Mouse Primary Material: Blood and organ isolation from mice, along with the subsequent in vitro staining protocols were used for authentication, and have been described in the Methods section. |
| Mycoplasma contamination | MCA205 cells were tested weekly using a PCR-based mycoplasma contamination assay.<br>All primary murine materials were handled under sterile conditions and used on the same day to ensure the absence of mycoplasma contamination. |
| Commonly misidentified lines<br>(See ICLAC register) | No commonly misidentified cell lines were used in the study. |

## Animals and other research organisms

Policy information about studies involving animals; ARRIVE guidelines recommended for reporting animal research, and Sex and Gender in Research

| | |
|---|---|
| Laboratory animals | Wild-type and TCR/HLA Transgenic Mice:<br>ABab-A2/ABabDII mice, as described by Li et al. (2010, Nat Medicine);<br>C57BL6/N mice, bred in-house at the MDC core facility in Berlin, Germany;<br>ABab-I mice, as described in this study. |
| Wild animals | n/a |
| Reporting on sex | Primary materials were obtained and analyzed from over 200 gender-mixed mice, with no concerns about gender-specific bias. For tumor antigen immunization, groups of 6-7 female mice were used per antigen, excluding other gender-mixed repetitions. |
| Field-collected samples | *For laboratory work with field-collected samples, describe all relevant parameters such as housing, maintenance, temperature, photoperiod and end-of-experiment protocol OR state that the study did not involve samples collected from the field.* |
| Ethics oversight | We were granted permission to conduct all mouse experiments including transgenic mouse generarion in this study by the Landesamt für Arbeitsschutz, Gesundheitsschutz und Technische Sicherheit, Berlin (Animal License Numbers: X9005/22, X9010/21, G-0111/17-16082, G-0111/17-16082-2, TVV-322/10), in accordance with the standard guidelines approved by the committee. |

Note that full information on the approval of the study protocol must also be provided in the manuscript.

## Plants

| | |
|---|---|
| Seed stocks | n/a |
| Novel plant genotypes | n/a |
| Authentication | n/a |

# Flow Cytometry

## Plots

Confirm that:

- [ ] The axis labels state the marker and fluorochrome used (e.g. CD4-FITC).
- [x] The axis scales are clearly visible. Include numbers along axes only for bottom left plot of group (a 'group' is an analysis of identical markers).
- [ ] All plots are contour plots with outliers or pseudocolor plots.
- [x] A numerical value for number of cells or percentage (with statistics) is provided.

## Methodology

| | |
|---|---|
| Sample preparation | For immunophenotyping, blood, spleen, and lymph nodes were freshly isolated under sterile conditions for immune T cell enrichment. A similar procedure was followed for anti-human HLA staining using primary materials from transgenic mice.<br>For repertoire sequencing, CD3+ CD8+ T cells from blood, spleen, and peripheral lymph nodes were enriched using flow cytometry sorting. |
| Instrument | All flow cytometers were from BD Biosciences GmbH, Heidelberg, Germany.<br>Flow Analyzers: FACSCelesta and FACSCanto II<br>Flow Sorters: FACSAria I. |
| Software | FlowJo software, version 10.7.1, TreeStar Inc., Ashland, Oregon, USA.<br>FACSDiva software, version 9.0, BD Biosciences GmbH, Heidelberg-Germany. |
| Cell population abundance | CD3+ CD8+ T cell populations were enriched using untouched magnetic beads and sorted by flow cytometry to achieve ≥ 97% purity. We isolated at least 300,000 cells from each sample. High-quality DNA was extracted from these samples for TCR repertoire deep sequencing. |
| Gating strategy | For all flow cytometry experiments, T cell populations were gated based on their morphology using FSC-Area vs. SSC-Area, with single cells delineated via FSC-Height vs. FSC-Area. Positive and negative delineation was based on standard T cell morphological characteristics and scatter separation. For ß2m stainings, boundaries were established using isotype controls, allowing for precise differentiation of ß2m-positive and -negative populations.<br><br>Supplementary Figures 2 and 4 have now been added to graphically depict the FACS sequential gating and sorting strategies used in the study.<br><br>Gating Strategy for T Cell Development Stainings (Fig. 2A)<br>Row 1: FSC-A vs. SSC-A was used to capture total thymocytes, which were then gated to display CD4 vs. CD8 populations.<br>Row 2: FSC-A vs. SSC-A captured lymphocytes, subsequently gated to show CD5 vs. CD69 populations.<br>Row 3: FSC-A vs. SSC-A captured lymphocytes; SSC-Height vs. SSC-A was used to isolate single cells, followed by SSC-A vs. CD3 to select CD3+ cells, which were depicted in histogram format.<br>Row 4: Similar to Row 3, FSC-A vs. SSC-A captured lymphocytes, followed by SSC-Height vs. SSC-A for single cell isolation, and SSC-A vs. CD3 for CD3+ selection, then showing CD4 vs. CD8 gating.<br><br>Gating Strategy for Splenic Lymphocytes (Fig. 2D).<br>Row 5: FSC-A vs. SSC-A captured splenic lymphocytes; SSC-Height vs. SSC-A isolated single cells, followed by SSC-A vs. CD3 for CD3+ selection and SSC-A vs. CD8 for CD8+ selection, depicting SSC vs. CD8+CD44high gating.<br>Row 6: Following the same initial gating as Row 5, splenic lymphocytes were processed to select CD3+ and CD4+ cells, depicting SSC vs. CD4+CD44high gating.<br><br>Gating Strategy for Lymphocytes (Fig. 3A)<br>FSC-A vs. SSC-A captured lymphocytes, followed by SSC-A vs. ß2m to delineate ß2m positives and negatives. The resulting populations were displayed as SSC-A vs. ß2m (+) and (−) based on isotype control.<br><br>Gating Strategy for Lymphocytes (Fig. 4)<br>Fig. 4A: FSC-A vs. SSC-A captured lymphocytes, followed by SSC-A vs. CD3 to select CD3+ cells, which were depicted to show CD4 vs. CD8 gating.<br>Fig. 4E: FSC-A vs. SSC-A captured lymphocytes, FSC-Height vs. FSC-A isolated single cells; FSC-A vs. 7-AAD to select live cells, followed by SSC-A vs. CD3 to select CD3+ cells, which were depicted to show CD4 vs. CD8 gating.<br><br>Gating Strategy for Lymphocytes (Fig. 10)<br>FSC-A vs. SSC-A captured lymphocytes; FSC-Height vs. FSC-A isolated single cells, followed by SSC-A vs. CD3 for CD3+ selection, which were depicted to show CD8 vs. IFN-γ gating.<br>The CD8+ IFN-γ+ populations, indicative of an antigen-reactive immune response, were gated based on long-term knowledge accumulated in the lab, as reported in previous works such as Li et al. (2010, Nat Med), Obenaus et al. (2015, Nat Biotechnol), Poncette et al. (2019, J Clin Invest), Gavvovidis et al. (2018, Clin Cancer Res), Immisch et al. (2023, Front Immunol), and Plewa et al. (2023, J Immunother Cancer). |

Gating Strategy for Lymphocytes (Supplementary Fig. 3A and 4A)

FSC-A vs. SSC-A captured MCA205 cells, followed by SSC-A vs. Aqua-Live/Dead Stain to select live cells. The resulting populations were displayed as SSC-A vs. six HLA-I monochains stained using pan HLA-ABC and ß2m antibodies. HLA-I (+) and (−) populations were delineated based on untransfected control.

☒ Tick this box to confirm that a figure exemplifying the gating strategy is provided in the Supplementary Information.
